# Supplementary material for: Membrane Proteomic Profiling of Soybean Leaf and Root Tissues Uncovers Salt-Stress-Responsive Membrane Proteins
Source: Int J Mol Sci. 2022 Oct 31;23(21):13270. doi: 10.3390/ijms232113270 (PMC9655375; doi:10.3390/ijms232113270)
Supplement: Supplementary file 1 [file ijms-23-13270-s001.zip › Supplementary Figures.pdf]

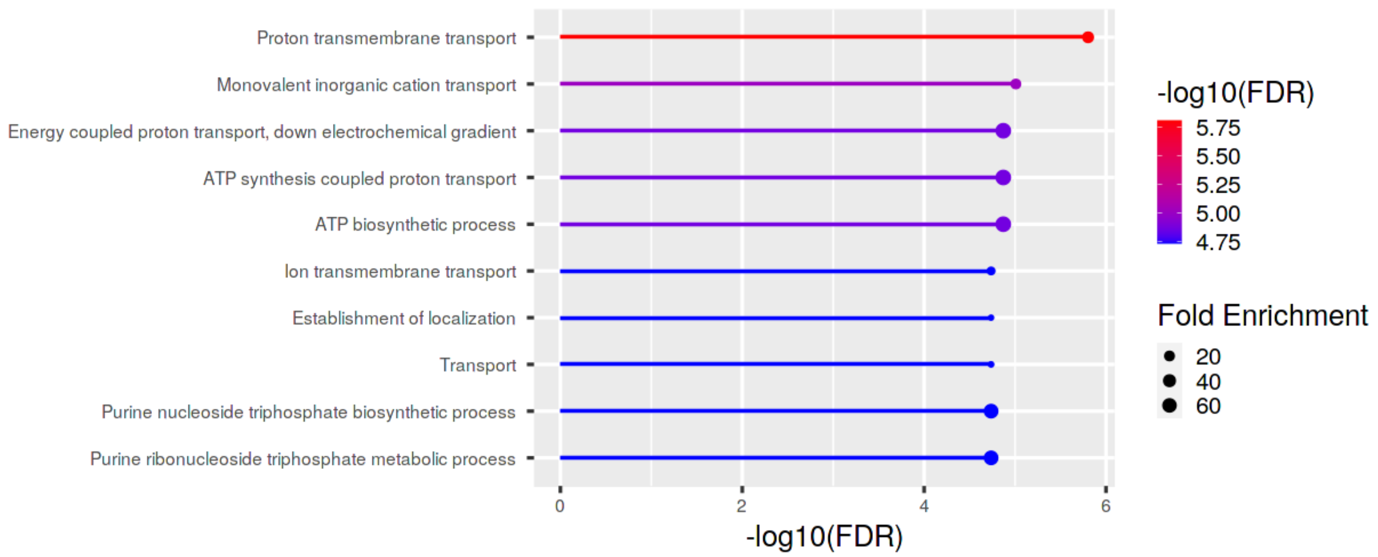
Proton transmembrane transport
Monovalent inorganic cation transport
Energy coupled proton transport, down electrochemical gradient
ATP synthesis coupled proton transport
ATP biosynthetic process
Ion transmembrane transport
Establishment of localization
Transport
Purine nucleoside triphosphate biosynthetic process
Purine ribonucleoside triphosphate metabolic process
0
2
4
6
-log10(FDR)
-log10(FDR)
5.75
5.50
5.25
5.00
4.75
Fold Enrichment
20
40
60

(a)

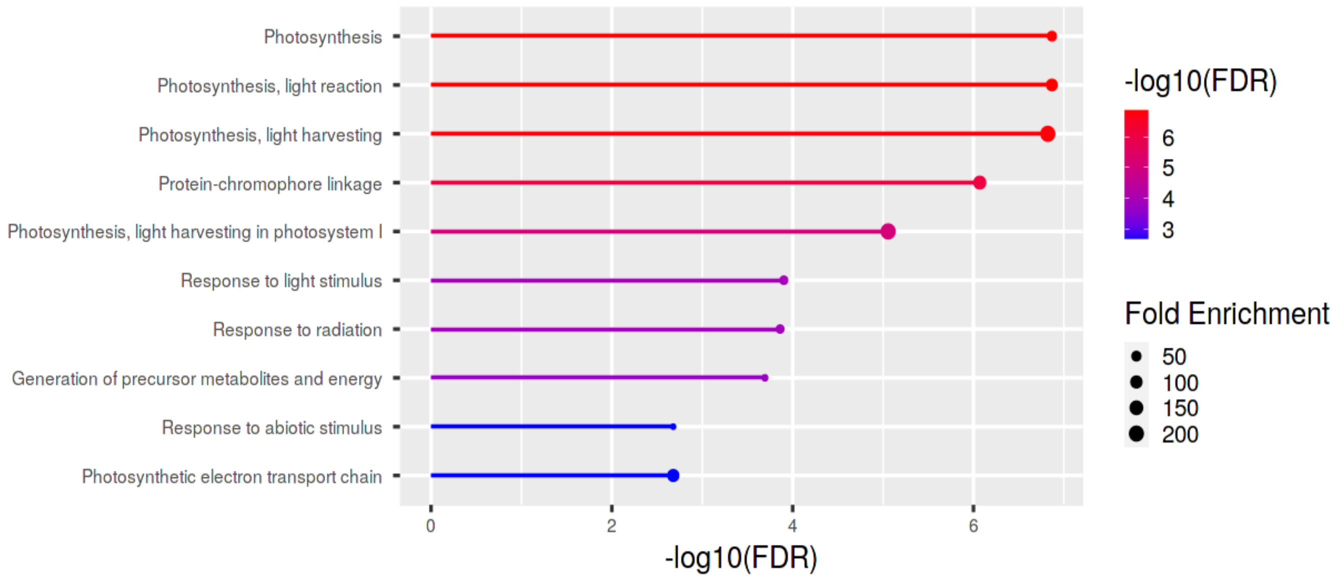
Photosynthesis
Photosynthesis, light reaction
Photosynthesis, light harvesting
Protein-chromophore linkage
Photosynthesis, light harvesting in photosystem I
Response to light stimulus
Response to radiation
Generation of precursor metabolites and energy
Response to abiotic stimulus
Photosynthetic electron transport chain
0
2
4
6
-log10(FDR)
-log10(FDR)
6
5
4
3
Fold Enrichment
50
100
150
200

(b)

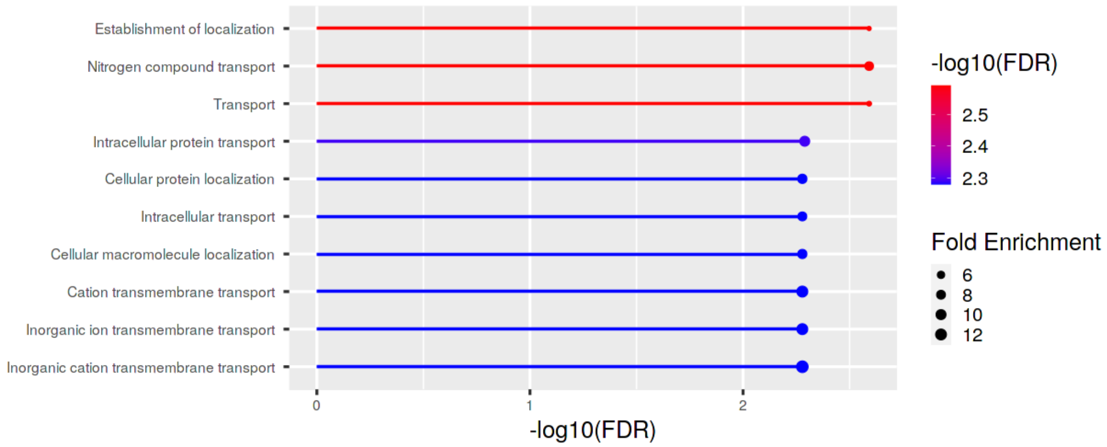

(c)

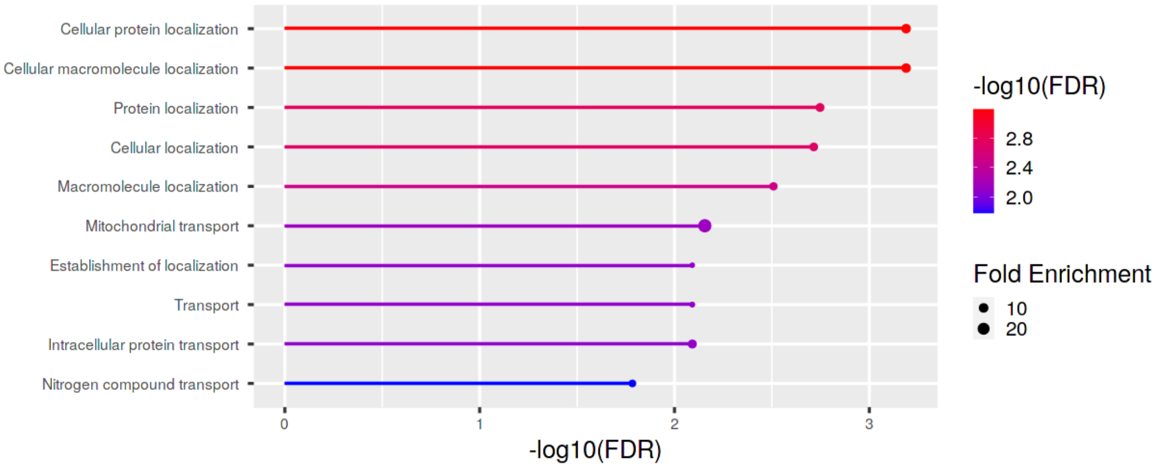

(d)

Figure S1: Molecular function Go enrichment of label free quantified membrane proteins in leaf and root tissues. (a) is showing the up-regulated membrane proteins functional enrichment analysis against salt stress in leaf. (b) is showing the down-regulated membrane proteins functional enrichment analysis against salt stress in leaf. (c) is showing the up-regulated membrane proteins functional enrichment analysis against salt stress in root. (d) is showing the down-regulated membrane proteins functional enrichment analysis against salt stress in root.

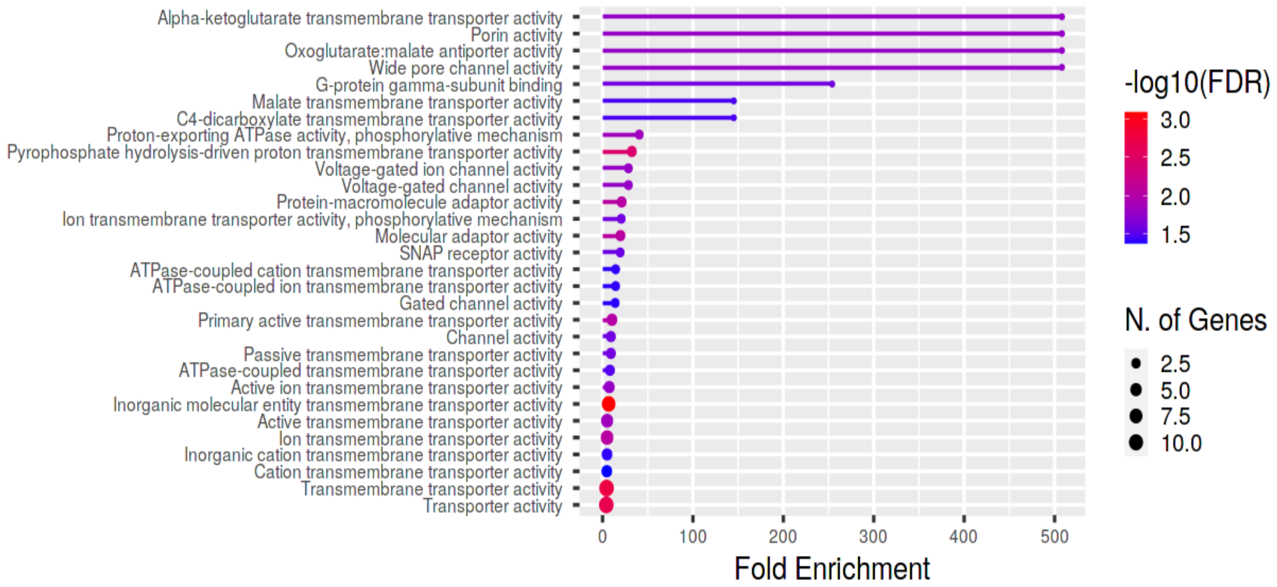

(a)

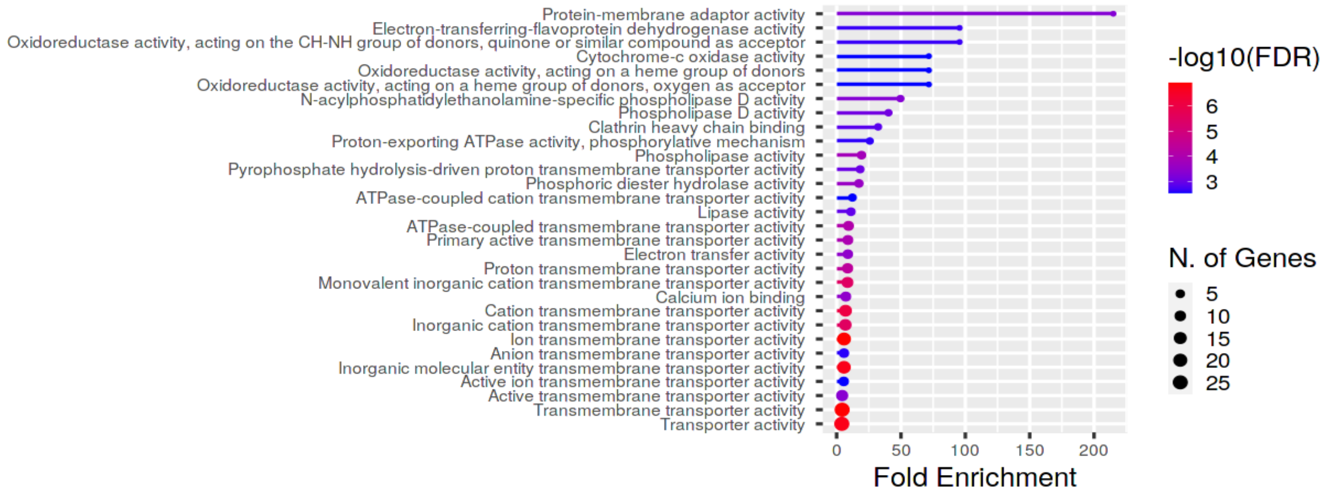

(b)

Figure S2: Molecular function Go enrichment of salt stress induced membrane proteins from leaf and root tissues. (a) is showing the molecular function associated terms of membrane proteins induced from leaf upon salt stress. (b) is showing the molecular function associated terms of membrane proteins induced from root upon salt stress.
